# Clinical pharmacology in Stockholm 50 years—report from the jubilee symposium

**DOI:** 10.1007/s00228-018-2432-6

**Published:** 2018-02-27

**Authors:** Michel Eichelbaum, Marja-Liisa Dahl, Folke Sjöqvist

**Affiliations:** 1 Dr. Margarete Fischer-Bosch Institute, Stuttgart and Division of Clinical Pharmacology, University of Tübingen, Tübingen, Germany; 2 Division of Clinical Pharmacology, Karolinska Institutet, Karolinska University Hospital, Stockholm, Sweden; 3 Division of Clinical Pharmacology, Karolinska Institutet, Stockholm, Sweden

On April 21st 2017, the 50 years’ jubilee of Clinical Pharmacology in Stockholm was celebrated at the Swedish Society of Medicine. Since the Department of Clinical Pharmacology at the Karolinska Institutet (KI) was among the first to be established worldwide, it is quite appropriate to reflect on its role for the development of the discipline with special emphasis on its work for the rational use of medicines (RUM).

Clinical pharmacology as an academic discipline started to develop in the late fifties and early sixties as a result of the so-called pharmacological revolution. At this time, many new drugs were introduced into the clinic which for the first time allowed the effective treatment of diseases such as hypertension, depression, schizophrenia, and many cancers to name a few. Many of these drugs came into clinical practice based on clinical trials in a limited number of patients using poorly defined clinical endpoints and scarce observations on adverse drug reactions. There were almost no data on their pharmacokinetics, the reason being that the analytical methods available at that time such as photometry, fluorometry, and thin layer chromatography were not sensitive enough to detect low blood concentrations. In these early days, it was not unusual that dose recommendations were based on the principle “One size fits all”. However, it was soon realized that the dose was a poor predictor of drug response, which triggered studies to ascertain the sources responsible for the large interindividual variability in drug response per unit dose. Hence, it became an urgent need to study the disposition and metabolism of these new drugs. Pharmacologists and analytical chemists in Stockholm were in the forefront in studying the disposition of new drugs. Instrumental for this development was the advent of modern analytical methods such as GC, HPLC, and particularly their combination with mass spectrometry, which for the first time allowed the specific and sensitive measurement of drug concentrations in the nanogram range. It was this pioneering work at KI in the mid-sixties that opened the doors for clinical pharmacological research. Right from the beginning, these modern analytical methods were not only seen as research tools but also used for therapeutic drug monitoring and thereby personalized medicine.

One of the main ideas of the organizers was to invite pioneer clinical pharmacologists and presently active clinical pharmacologists to jointly address some key questions in RUM. This report summarizes the presentations given at the symposium, which was supported by the Swedish Foundation for Clinical Pharmacology and Pharmacotherapy.

## The beginning of clinical pharmacology in Stockholm

The scientific program was started by one of the organizers (Folke Sjöqvist), who described the critical steps in the introduction of clinical pharmacology in Swedish medicine. Key persons who already in the late fifties realized the importance of getting access to professional pharmacology in health care included two professors in basic pharmacology Börje Uvnäs, the first president of IUPHAR, and Bo Holmstedt, one of the pioneers in mass spectrometric drug analysis as well as the influential internist Lars Werkö, once chairman of the Swedish Medical Association.

The first position as teacher in clinical pharmacology combined with consultancy in the university hospital was founded in Stockholm 50 years ago (1967) and followed by similar positions at all medical schools in Sweden. The first positions as full professors with responsibilities for research, teaching, and health care were later created in Linköping (1970) and Stockholm (1972). Legitimacy in clinical pharmacology was introduced in 1980 and the full specialty 10 years later. In 1990, all medical faculties had professorships in clinical pharmacology.

The further development of clinical pharmacology in Stockholm was not restricted to the university hospitals at the KI campuses in Solna and Huddinge, but positions as head physicians were later created in the two teaching hospitals South Hospital and Danderyd Hospital. The CP functions were focused on services to clinical colleagues in the form of drug evaluation and information often within the frame of the Drug and Therapeutics Committee (DTC), consultations and pharmacotherapeutic advice to clinical colleagues as well as interpretation of drug analyses from the therapeutic drug monitoring (TDM) laboratory. Research collaboration was encouraged and made up a powerful partnership with clinical colleagues. One example is the large clinical study on low-dose ASA after stroke (the SALT trial) that was monitored by the late Carl-Eric Elwin, the clinical pharmacologist at the Danderyd Hospital at the time. Among successful novelties should also be mentioned that the South Hospital had the privilege to host the first general practitioner doing academic outreach within clinical pharmacology (Lars-Olof Hensjö). The model of continued education about drug treatment that he led and performed, often together with a clinical pharmacologist or a pharmacist, eventually created followers in all Swedish counties and DTC:s.

The opportunities and difficulties for a lonely pharmacologist in a huge hospital were presented by the pioneer head physician at South Hospital, Rune Dahlqvist.

## Drug and therapeutics committees (DTC:s)

The second subject in the symposium was devoted to the importance of DTC:s for the development of clinical pharmacology in health care and presented by Lars L Gustafsson and Marie-Louise Ovesjö. The first DTC in Sweden was formed at Karolinska Hospital already in 1961 with the primary function to evaluate new and old drugs and to be responsible for the recommended assortment of drugs in the hospital. This function depended on smooth collaboration between clinicians, clinical pharmacologists, and pharmacists. After the demonstration by pharmacologists at the Serafimer hospital (the late Anders Rosén and Björn Beermann), that some frequently used anticholinergic drugs were not even absorbed the need to study the disposition of drugs in humans and the need to involve clinical pharmacological expertise in drug evaluation became obvious. During the years to come, the Swedish system of regional DTC:s grew rapidly and became the most important working place for clinical pharmacology [1].

The list of recommended drugs in Stockholm is now called the Wise List and is published in two versions, one for prescribers (mainly physicians) and one for patients [2]. The adherence in primary care to the recommendations in the Wise List has increased from 80% in 2005 to 90% in 2015. It was suggested that the major reasons for the high adherence to the recommendations were the multifaceted approach with collaboration between respected local pharmacotherapeutic experts, clinical pharmacologists, and pharmacists combined with extensive communication and marketing (even in the underground trains!) of the Wise List and the good access to continuous medical education [3]. This approach is known in Scandinavia as the Stockholm Model for the Rational Use of Medicines.

The DTC:s also provide drug information and postgraduate teaching in therapeutics for all personnel in health care. In 2016, the DTC in Stockholm together with clinical pharmacology arranged several hundred meetings for continued drug education attended mainly by physicians and nurses.

## Pharmacoepidemiology

The development of pharmacoepidemiology was triggered by the thalidomide disaster in 1961 which lead to two public health registers in Sweden on fetal malformations (1964) and adverse drug reactions (1965), respectively. Clinical pharmacologists from Stockholm have played a decisive role in the development of these registers, with Barbro Westerholm as adjunct professor in pharmacoepidemiology from 1979. Her first pupil, the late Bengt-Erik Wiholm, introduced pharmacoepidemiological thinking both in academia and in drug control in Sweden, particularly regarding the recording of adverse drug reactions.

Barbro Westerholm, Ulf Bergman, and Björn Wettermark told how they simplified the measurement of drug utilization by introducing the terms DDD and PDD (defined and prescribed daily doses) as well as DU 90% (drug utilization covering 90% of the used volume). Hereby, the doses used rather than drug names came in focus [4].

Fundamental for the growth of drug utilization research in Sweden was the establishment of the Swedish Prescribed Drug Register in July 2005. This register contains patient-level data for all prescription drugs dispensed in Sweden. Pharmacoepidemiology has developed rapidly in Sweden during the past 50 years with the increased access to registers and advanced analytical methods to adjust for confounding. However, it should be emphasized that the focus of inquiry is still very much the same as the original ideas raised by pioneers in the early days of clinical pharmacology.

## Clinical pharmacology in drug control

The roles of clinical pharmacology in drug control and postgraduate drug information emerging from the Swedish Medical Products Agency were analyzed by two senior clinical pharmacologists (Gunnar Alván, Björn Beermann) and a presently active colleague (Charlotte Asker-Hagelberg). During the years, excellent relations between academic and regulatory clinical pharmacology have developed in Sweden. Gunnar Alván pointed out that the duties of the Swedish Medical Products Agency and academic clinical pharmacology are “like made for each other”: drug evaluation, drug safety, rational use of medicines, and drug education [5]. Most senior clinical pharmacologists have been involved in the successive improvement of the quality of clinical trials by taking responsibility for advanced educational programs.

About 20 clinical pharmacologists, most of them trained in Stockholm, have been appointed within the drug control agency. Two academic clinical pharmacologists have become Directors General of the Swedish drug control (Kjell Strandberg and Gunnar Alván), and one (Barbro Westerholm) has served as Director General of The National Board of Health and Welfare.

## Introduction of new drugs in health care

The next two subjects were the principles of drug evaluation and managed introduction of new drugs in health care. Paul Hjemdahl pointed out that critical drug evaluation is a natural task for clinical pharmacologists, who should be well trained in pharmacodynamics, pharmacokinetics, and therapeutics as well as in clinical trial methodology (design, analysis, interpretation), and preferably also be well versed in pharmacoepidemiology and health economy. In this work, the clinical pharmacologist should avoid conflicts of interest with industry, and be well acquainted with pharmaceutical marketing techniques.

Two examples presented were the weight loss drug rimonabant which was launched as a wonder drug to treat the metabolic syndrome [6], and the novel, direct-acting oral anticoagulants (NOACs) as alternatives to warfarin for stroke prevention in atrial fibrillation (AF) [7].

Rimonabant received skeptical comments due to relatively poor efficacy, many drop-outs in the pivotal trials and because the drug (a cannabis CB_1_-receptor antagonist) was associated with psychiatric side effects which precluded use in patients requiring psychotropic drugs. The evaluators in Stockholm found that the effectiveness of rimonabant was even worse than in the trials and that one fourth of the patients on rimonabant also had taken antidepressants, contrary to the recommendations. The drug sold poorly and was soon taken off the market.

Work with the introduction of NOACs commenced with analyses of all AF patients in the Stockholm County, including those in primary care, in the pre-NOAC era. Only half of them received warfarin treatment and less efficient aspirin therapy was common, especially among the many elderly (1/3 were 80 years or older and many of them were frail). The largest stroke burden was found among elderly patients who were not treated with warfarin. The pivotal NOAC trials in AF were critically evaluated from the Swedish perspective, and it was found that the quality of warfarin treatment was suboptimal (giving the NOACs a larger advantage than was reasonable in our region) and that few elderly patients were included. Marketing messages about the simplicity of NOAC treatment were exaggerated.

The DTC launched an ambitious educational program about NOACs before including them in the Wise List in 2015. The follow-up of treatment results in Stockholm showed that the NOACs were at least as effective and safe as warfarin in patients including those 80 years and above and patients with previous bleeds. Observational studies from Scandinavia and the USA reached similar conclusions, and in 2017, we gave apixaban priority before warfarin or dabigatran in the Wise List.

Rickard Malmström reported about the Stockholm model for managed introduction of new medicines. In the early 2000s, clinical pharmacology in collaboration with the Stockholm DTC was instrumental in building and launching a model starting with horizon scanning, a process where new medicines in pipeline are identified before market approval. The purpose is to prepare the health care system before the introduction of new drugs based on critical appraisal, early recommendations about the use and follow-up, and handling of drug budget consequences.

In 2012, clinical pharmacology in collaboration with the hospital board launched a model for managed introduction of new drugs at Karolinska University Hospital. The clinic applies for introduction of a new drug to the hospital medicines council detailing estimated number of patients, cost, priority by the national pharmaceutical benefits agency (TLV), or when applicable by the new therapies (NT) council of the Swedish county councils, and Stockholm DTC, together with a treatment decision protocol describing the clinical indications.

## Drug information services

The experiences of the first hospital-based drug information center in Sweden (Karolic) established in 1974 and chaired by Gunnar Alván for 25 years at Huddinge were reviewed by Annika Asplund, Birgit Eiermann, and Ylva Böttiger. They reported about the successive increase of sophisticated questions from the health care, particularly from clinical colleagues and about the development of electronic data bases.

From the very beginning, the working method was critical evaluation of the scientific literature, based on the experience in the National Library of Medicine in Bethesda, where the first chief consultant to the center, the late Carl-Eric Elwin, was trained. The center is staffed by pharmacists and clinical pharmacologists working together to solve patient-related drug problems, particularly regarding efficacy, adverse effects, drug interactions (DDI:s), and drugs in pregnancy and during nursing. The working philosophy of the center has been presented in the Lancet [8]. Selected evaluations of the incoming questions are regularly published in the Swedish Medical Journal, thus contributing to postgraduate drug education.

The quality of drug information services in the Scandinavian countries was assessed recently comparing seven drug information centers responding on 718 questions from GP:s under blind conditions. The questions needed in average 3 h to be handled with variations from a few to 600 min showing the complexity of most of the questions received. One of the key quality criteria was that the answers include concrete advice to the clinician, not only evaluation of the available evidence.

Already 1970 the Swedish drug industry and the Swedish Drug Regulatory Agency invited clinical pharmacologists at KI to review existing DDI:s in the physicians’ desk reference book [9]. Their chapter has since then been updated yearly, and its classification was used in the digital drug-drug interaction database, SFINX (Swedish Finnish Interaction X-referencing), initiated by the so-called Janus project for providing drug information and decision support at point of care [10]. The SFINX drug-drug interaction database was developed in a collaboration between Swedish and Finnish clinical pharmacologists starting in 2003 and released in the two countries in 2005 [11]. From 2007, SFINX was released on the website of the Stockholm County Council (www.janusinfo.se) and through the Janus toolbar in electronic patient record systems. Since 2010, SFINX is distributed nationwide and widely used even outside Sweden and Finland. In 2016, even the Swedish pharmacies decided to integrate SFINX into their drug dispensing systems.

The impact of integration of SFINX into primary health care records on the prevalence of DDIs has been studied in 15 primary health care centers [12]. Use of SFINX was associated with a 17% decrease, from 2.15 × 10^3^ to 1.81 × 10^3^ interactions per prescribed drug-drug pair, in the prevalence of potentially serious D-interactions.

Since 2017, SFINX is called Janusmed Interactions and complemented by another database called “Janusmed risk profile”. The latter contains grading of all substances with regard to nine clinically relevant pharmacodynamic effects such as risk of bleeding, postural hypotension, QT-prolongation, sedation, or anticholinergic effects. Using algorithms, a risk grade is presented for any drug combination prescribed to a patient.

Several other electronic knowledge databases focusing on drugs and pregnancy, drugs and breast feeding, drugs and gender/sex, a pediatric database ePed, drugs and the elderly and the side effect database “Bikt”, all aiming to facilitate RUM, have been developed in close collaboration between clinical pharmacologists, pharmacists, pharmacotherapeutic experts, epidemiologists, and IT experts.

## Obstetric and pediatric pharmacology

Clinical pharmacological aspects in obstetrics and pediatrics were discussed by Anders Rane. Pediatric clinical pharmacology in Stockholm started with the early discovery (1970) at Karolinska Hospital that human fetal liver in contradistinction to the conditions in animals contains functional cytochrome P450. The late Lars-Olof Boréus was one of the senior authors [13] and became later (1997) the first professor in pediatric pharmacology at KI. Anders Rane was the junior investigator in these studies and now, almost 50 years later, he summarized pharmacokinetic studies in pregnant women and children performed in Stockholm.

The most conspicuous finding was the 4.4-fold increase of apparent oral clearance of metoprolol in pregnant women compared to the post-pregnancy values [14]. This was because CYP2D6 was shown to be uniquely induced by the “endocrine environment” of the pregnant woman. The research group subsequently confirmed the effect of pregnancy on CYP2D6 using dextromethorphan as probe drug.

Specifics of PK and PD in neonates and children have been subject to numerous publications of the group. The perinatal switch in metabolic pattern was studied in human fetal hepatocytes [14] with acetaminophen as substrate—from prenatal glutathione conjugation and sulfation to postnatal glucuronidation [15]. The neonatal immaturity in metabolic elimination of different drugs has been studied with oxazepam and phenytoin. In contrast, the clearance of many medicines was shown to be increased in the toddler and early school age, e.g., carbamazepine [16] which underlines the importance of TDM in pediatric medicine.

A novel knowledge database—ePed—for safe drug selection, administration, and dosage in children has been developed at Karolinska University Hospital and was presented by one of the innovators, Synnöve Lindemalm. “e” in ePed stands for evidence based, experience based, and electronic drug information. The ePed (www.eped.se) concept includes not only the database but also a multiprofessional working group consisting of pediatricians, clinical pharmacologist, pharmacist, and pediatric nurse and a national network of pediatric hospitals.

Anders Rane also presented the current establishment of networks in the Nordic countries, and their collaboration with many EU countries (Pediatric Clinical Research Infrastructure Network, www.ecrin.com) aiming to promote the development of drugs for children and to create sites of excellence across Europe for that purpose.

## Geriatric pharmacology

Johan Fastbom and Pauline Raaschou reported several measures initiated by clinical pharmacology designed to avoid polypharmacy and eminently unsuitable drugs in the elderly, systems that now are in routine use in Swedish health care.

On behalf of The Swedish National Board of Health and Welfare geriatric pharmacologists in the early 00:s developed indicators for the evaluation of the quality of drug use in the elderly. They were released for the first time in 2004 and in a revised form 2010. The indicators have given rise to several initiatives and received a variety of applications in the care sector. These include educational initiatives for healthcare professionals in the field of drugs and aging; recommendations and quick reference guides for drug treatment in old age; support of drug utilization reviews, regular national measurements and comparisons of quality of drug use in the elderly, and initiatives from pensioners’ organizations, aiming to educate patients towards improved drug treatment.

Since 2005, there are clear signs of improvements in older people’s use of medicines in Sweden. For example, the latest nationwide measurements from the National Board of Health and Welfare show that the use of generally inappropriate drugs, NSAIDs, and antipsychotics has decreased by 53, 51, and 43%, respectively, in people 75 years and older, between 2005 and 2016 [17]. The Swedish national indicators for quality of drug therapy in the elderly have thus improved RUM in older people. The indicators have recently been thoroughly revised and a new version was released in June 2017.

Pauline Raaschou described how geriatric pharmacology has successively gained recognition in the DTC organization and activities. The chapter on drugs and aging in the Wise List, first introduced in 2005, has by now developed to include drug recommendations in diagnoses common in the older patients such as depression, sleeping disorders, anxiety, and dementia. The focus is also increasingly on “frailty” or “unsuccessful aging” instead of aging as such.

## Drug analysis

In contradistinction to most other European departments of clinical pharmacology, KI has prioritized the development of therapeutic drug monitoring (TDM) basing the service on excellent collaboration between pharmacologists and analytical chemists. The approach is much inspired by the school of BB Brodie at NIH. The TDM laboratory in Stockholm is now one of the most diversified in Europe. The development of the laboratory services was reviewed by the analytical chemists Olof Beck, Anton Pohanka, and Tomas Villén. They pointed out that drug analysis has long traditions at KI and mentioned in particular the publications in 1968 by Bo Holmstedt and collaborators [18], some later becoming involved in clinical pharmacology. These publications concerned massfragmentography for measuring for the first time very low blood levels of chlorpromazine and tricyclic antidepressants [19]. This technique permits a coupling interface between gas chromatography and mass spectrometry. Other important analytical techniques have been immunochemistry and liquid chromatography. Since the late nineties, the use of liquid chromatography-mass spectrometry has allowed for the measurement of drugs and metabolites including glucuronides which could not be measured by GC-MS. The recent access to LC-high resolution MS offers unique possibilities to get the whole drug picture in the patient (several drugs measured simultaneously).

**Fig. 1:**
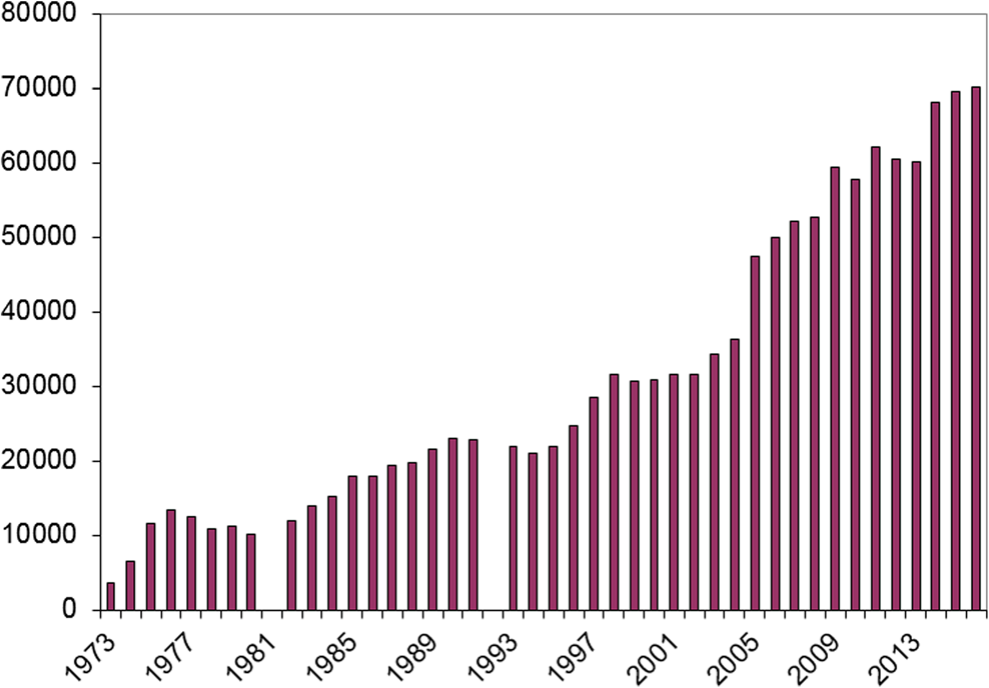
Number of TDM-measurements 1973–2016

**Fig. 2:**
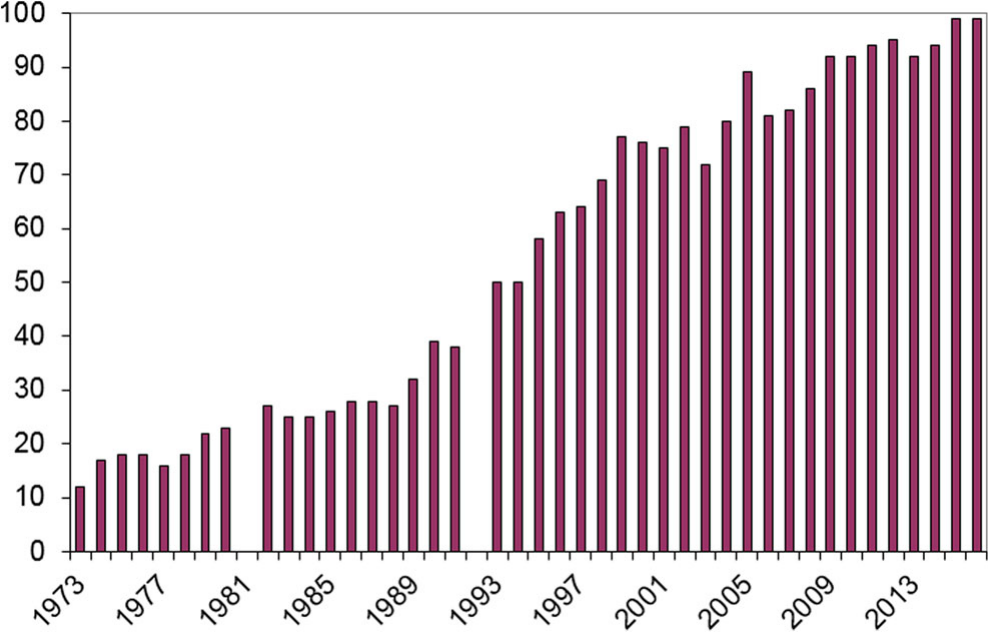
Number of different TDM-assays 1973–2016

**Fig. 3:**
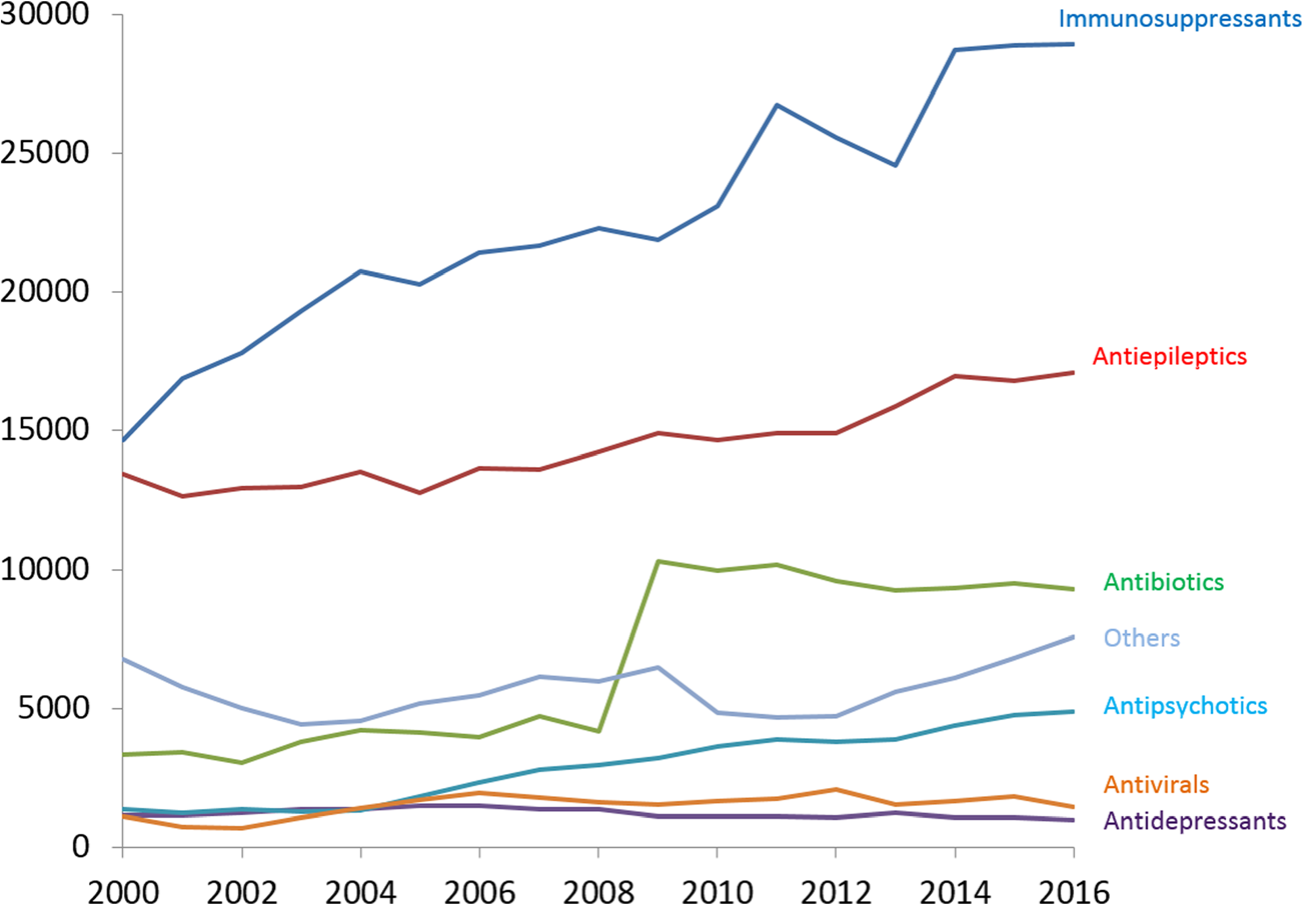
Number of TDM-requests per major therapeutic area 1973–2016

The number of analyses and the spectrum of drugs analyzed by the TDM laboratory has increased steadily over the years (Figs.1 and 2). In the early seventies, a dozen drugs were analyzed and the number of analyses was a few thousand yearly. In 2016, more than 70,000 samples were analyzed of nearly 100 different drugs. The largest groups of drugs are presently immunosuppressives, antiepileptics, and antibiotics (Fig. 3). In addition, the lab handles 140,000 biological samples for drugs of abuse. The laboratory is accredited by IOC for doping analyses as well. To this comes an increasing number of genotypic and phenotypic characterization of TPMT and CYP enzymes (mainly CYP2D6, CYP2C9, CYP2C19, and CYP3A5).

The availability of a substantial TDM-database has facilitated a diversified DDI-research. A dozen drug interactions has been discovered.

## Therapeutic drug monitoring

The potential benefits of TDM in improving drug therapy were critically reviewed for psychiatry (Marja-Liisa Dahl and Jonatan Lindh), neurology (Torbjörn Tomson), antibiotics (Erik Eliasson, Jaran Eriksen), oncology (Alan Fotoohi and Curt Peterson), and transplantation/immunosuppression (Staffan Rosenborg and Bo-Göran Ericzon).

Marja-Liisa Dahl and Jonatan Lindh spoke on individualized pharmacotherapy based on TDM and pharmacogenetics and gave examples from psychiatry. Early pioneering work at KI established already in the late 1960s the large, more than 30-fold interindividual differences in the steady-state plasma concentrations of the tricyclic antidepressant (TCA) nortriptyline given in fixed doses. Using panels of mono- and dizygotic twins, it was shown that this variability was influenced both by genetic and environmental (drug interactions) factors [20]. TDM was recognized already in the early 1970s as a valuable tool for monitoring treatment of depression with TCAs.

In parallel with this, the role of the cytochrome P450 (CYP) enzyme system, and in particular the polymorphic CYP2D6, for the metabolism of antidepressant and antipsychotic drugs was identified, explaining part of the interindividual variability in pharmacokinetics and dose requirement. Much of the early work was focused on poor metabolisers tolerating only low doses of TCAs. Case reports of very fast metabolisers of antidepressants requiring extremely high doses for therapeutic effect later got their explanation when ultrarapid metabolisers of CYP2D6 with duplication or multiduplication of the active gene were discovered in the early nineties, in collaboration with the laboratory of Ingelman-Sundberg [21].

Measurements of antidepressant and antipsychotic drugs combined with genotyping of CYP2D6 and CYP2C19 are now an important part of the TDM service in Stockholm [22].

Torbjörn Tomson presented a thorough analysis of the use of TDM in the treatment of epilepsy. Phenytoin was the first antiepileptic drug to be monitored, already in the early sixties when discussions started about the need to individualize the dosage mainly because of its concentration dependent kinetics. So-called therapeutic ranges of plasma levels of antiepileptics were introduced as guidelines for rational dosing.

The most convincing studies have been performed with the old drugs, in particular phenytoin [23] while too many studies of newer compounds have been less convincing mainly due to their poor design. Given the challenges in concentration-effect studies in epilepsy, trigeminal neuralgia has also been used as a model in studies of carbamazepine [24].

The International League Against Epilepsy has introduced the concept “individual optimal/therapeutic serum concentration” as a more useful guideline than general “therapeutic concentrations”. This individual concentration is determined by monitoring of the drug during seizure-free periods and may be used as a reference value at therapeutic failure, pregnancy, and potential risks for drug interactions [25].

The importance of TDM for optimal immunosuppression was reviewed by Bo-Göran Ericzon and Staffan Rosenborg. The collaboration between the transplantation clinic and clinical pharmacology has been intense since the introduction of cyclosporine A. This has resulted in 45 joint publications and several PhD-theses. Immunosuppressant drugs fulfill most criteria that make TDM meaningful and facilitate the understanding of relationships between plasma concentrations, effects, and side effects [26].

The analytical methods have successively improved to include both parent drugs and metabolites. Presently, 30,000 samples are analyzed yearly and commented upon by a clinical pharmacologist within the same working day.

TDM of anti-infective agents was reviewed by Erik Eliasson and Jaran Eriksen. It is unique in the sense that the target sensitivity (i.e., required drug concentrations for antimicrobial effect) may be better understood in the individual case than in most other therapeutic areas. Therefore, microbiology diagnostics combined with TDM offers a quite powerful tool in personalized medicine. This has been recognized by clinicians, and both the number of different anti-infective agents to monitor and the overall volume of TDM-requests show a steady increase over the last decades. The early examples in this field have been drugs with significant risk of toxicity at high exposure levels, like aminoglycosides and glycopeptides. Even if drug toxicity is still the main clinical reason for TDM in this therapeutic area, including more recent classes of anti-infective agents like triazole antifungals and herpes antivirals, there is now increased awareness of the risk of sub-therapeutic exposure levels, especially in the critically ill within intensive care or in patients with more chronic infection [27]. Risks associated with low exposure levels do not only relate to impaired clinical efficacy, prolonged hospitalization, and even increased mortality, but also the risk for the development of antimicrobial resistance [28].

In conclusion, monitoring of anti-infective agents is meeting an increased interest among clinical colleagues and in particular for its relevance in the care of critically ill patients. The overwhelming threat of antimicrobial resistance will further support the use of individualized treatment regimens, for which TDM will be an essential tool in dose optimization.

Curt Peterson and Alan Fotoohi reported about the increasing interest for TDM in oncology. Plasma concentration monitoring of methotrexate has long been mandatory in high-dose treatment of childhood leukemia and osteosarcoma for dose adjustment of leucovorin, an antidote of methotrexate. Another well-established area is monitoring of thiopurines in maintenance treatment of childhood leukemia and also in inflammatory bowel disease. TPMT is a polymorphic enzyme and reduced activity shifts the metabolism from less active methylated metabolites to more active phosphorylated metabolites increasing the risk of severe adverse reactions. It is now clinical routine in Sweden to do pheno- and/or genotyping of TPMT before initiation of therapy for dose determination. Furthermore, it is well established to monitor phosphorylated and methylated metabolites during treatment for dose adjustments.

In the past, cancer chemotherapy has most often been given as intravenous infusions once every 3 weeks. Dose modifications (reductions) have been based on clinical toxicity. No logistics has been developed for dose increases. Progress in the understanding of cellular processes regulating cell growth, differentiation, and death has led to the development of drugs inhibiting crucial steps in cell growth often tyrosine kinase inhibitors. These drugs are given orally in daily doses so that steady-state concentrations are reached. One of the first drugs of this type is imatinib which revolutionized the treatment of chronic myeloid leukemia.

Alan Fotoohi reported on pharmacokinetic studies showing large interindividual variability of 5-fluorouracil (5-fu) and imatinib. TDM-based dose adjustment of 5-fu has been shown to significantly reduce the risk of severe toxicities and prolong progression-free survival in patients with metastatic colorectal cancer [29]. TDM-based dose optimization intervention of imatinib improves the response [30]. However, more efforts are needed to present the evidence indicating advantages of TDM of 5-fu and imatinib and make them routine approaches in oncology and hematology. There is growing evidence indicating that TDM can be beneficial for some other anticancer agents, for instance paclitaxel, busulfan, sunitinib, and tamoxifen. Another example is erlotinib, an inhibitor of epidermal growth factor receptor used in the treatment of lung cancer. Pharmacokinetic studies have shown much higher clearance in smokers probably as a result of induction of CYP1A2. However, this is not taken into consideration in routine drug dosing.

So far, oncologists have not been used to take advantage of TDM approaches, and drug dosing in clinical practice is based on pivotal clinical trials. There is a tendency both in USA and Europe for early approval of cancer drugs based on less robust trials not taking due consideration to optimal dosing and interindividual variability. We believe that TDM will be more and more used in oncology in coming years [31].

## Treatment of pain—the clinical pharmacological aspects

Anders Rane presented the comprehensive opioid research that has been performed in Stockholm. Early consultation requests in the 1970s–1980s from Surgery, Oncology etc. asked for advice for oral treatment of persistent severe pain in patients since frequent parenteral administration was untenable in the long run. The Swedish National Board of Health and Welfare stated at that time that “oral preparations of morphine were of doubtful benefit because of their low and inconsistent bioavailability”. The research group performed extensive PK studies of oral morphine in cancer patients with severe pain [32, 33]. It was demonstrated that full pain relief can be achieved if oral doses were high enough. The oral bioavailability was shown to be around 38% (range 15–64%), and no metabolic tolerance was observed despite extensive dose increases over time. The Swedish PDR did not include any oral preparation of morphine in 1982. The following year, there was one marketed oral morphine preparation in the Swedish PDR, compared with a dozen pharmaceutical preparations in 2015. This is an illustrative example how collaborative clinical research can improve treatment of severe pain.

Later in the symposium, the evaluation of analgesic drugs within the DTC in Stockholm was described by Carl-Olav Stiller in a 20 year’s perspective. He gave examples of recent changes in the routine clinical use of COX-2 inhibitors and opiates based on academic drug evaluations. In the examples, critical analysis of the literature resulted in a comeback of the “old” naproxen on the list of recommended drugs and a decline in the use of the “modern” tramadol. In a few instances, the DTC had to design its own clinical trial to reach the correct cost-benefit appraisal of the drug and the right balance between commercial and academic drug information.

## Teaching of clinical pharmacology

The final part of the symposium was devoted to the present teaching of personnel in health care. Georgios Panagiotidis and Eva Wikström Jonsson reported about their unique experience to teach different professional groups with the same ambition to provide rational drug therapy. The teaching of clinical pharmacology for medical students at KI was introduced in the sixties. Central topics during these years have been drug evaluation, variability in kinetics and effect, prescribing skills, pain relief, long-term prevention of cardiovascular disease, and drug therapy in renal dysfunction. Prescribing skills are practiced in seminars held jointly by clinical pharmacologists and general practitioners. Seminars are based on patient-cases or scientific articles. Some of the seminars are integrated with geriatrics. Clinical pharmacology is examined in a small separate exam and as part of internal medicine and the integrated final. Focus is on principles for effective and safe drug treatment, individualized (personal) treatment, adverse effects and interactions, clinical trials, and critical evaluation of studies and drug information. During the last two semesters, last year students on the MD-program at KI have been able to choose a 5-week course in clinical pharmacology as an elective. In 1998, the Stockholm University College of Health Sciences merged with KI, bringing with it several new study programs, e g, midwifery, biomedical laboratory science, dental hygienist, nursing, and specialist nursing programs. In all study programs, there are courses in basic and clinical pharmacology and clinical pharmacology is the course provider.

Another key role for clinical pharmacology is to offer independent drug education and sources of information and to provide continuous medical eduation in critical drug evaluation. Clinical pharmacology is a resource providing scientific background, teaching material, and outreach visits for general practitioners and nurses as well as specialists in hospitals and specialist centers.

By all these activities, clinical pharmacology contributes to RUM by educating many different categories of healthcare professionals.

## Closure of the symposium

At the very end of the symposium, the organizers summarized some golden rules for the success of clinical pharmacology in the future:

Focus the main activities in teaching, research, and services on the principles in the rational use of medicines.

Prioritize research collaboration with clinical disciplines.

Acquire broad general knowledge in pharmacotherapeutic principles rather than competence restricted to a single area.

Take a leading role in drug education of all health care personnel.

Get pharmacopolitical visibility in the society.

Finally, Folke Sjöqvist advised his colleagues:

DO NOT ASK WHAT CLINICAL PHARMACOLOGY CAN DO FOR YOU BUT RATHER WHAT YOU CAN DO FOR CLINICAL PHARMACIOLOGY IN THE SENSE OF RATIONAL USE OF MEDICINES
